# Nationwide molecular epidemiology of methicillin-resistant *Staphylococcus aureus* responsible for horse infections in France

**DOI:** 10.1186/s12866-016-0924-z

**Published:** 2017-05-03

**Authors:** François Guérin, Marguerite Fines-Guyon, Pierrick Meignen, Géraldine Delente, Caroline Fondrinier, Nancy Bourdon, Vincent Cattoir, Albertine Léon

**Affiliations:** 1 Université de Caen Normandie, EA 4655 (équipe “Antibio-résistance”), F-14032 Caen, France; 2 CHU de Caen, Service de Microbiologie & CNR de la Résistance aux Antibiotiques (laboratoire associé “entérocoques et résistances particulières des bactéries à Gram positif”), Av. Côte de Nacre, 14033 Caen, Cedex 9 France; 3 Université de Caen Normandie, IUT département STID, F-14033, Caen, France; 4 LABÉO Frank Duncombe, F-14053, Caen, France; 5 CHU de Rennes, Service de Bactériologie-Hygiène hospitalière, Hôpital Pontchaillou, 2 rue Henri Le Guilloux, 35033 Caen, Cedex 9 France

**Keywords:** MRSA, Equine infections, ST398, ST8

## Abstract

**Background:**

The epidemiology of methicillin-resistant *Staphylococcus aureus* (MRSA) isolated in horse infections is not well documented, especially in France. The aim of the study was to evaluate the prevalence of MRSA isolates in horse infections from 2007 to 2013 in France and to characterize phenotypically and genotypically this collection.

**Results:**

Out of 1393 *S. aureus* horse isolates, 85 (6.1%) were confirmed to be MRSA. Interestingly, the prevalence of MRSA significantly increased from 2007–2009 to 2010–2013 (0.7 vs. 9.5%, *P* <0.0001). Resistance to methicillin was due to the presence of the *mecA* gene in 84 strains (98.8%) while one strain (1.2%) possessed the *mecC* gene. The vast majority of the strains (83/85, 97.6%) was resistant to at least three different classes of antibiotics. Multi-locus sequence typing (MLST) showed that MRSA strains belonged mainly since not all belong to two sequence types (STs): ST398 (53/85, 62.4%) and ST8 (28/85, 32.9%). It is worth to note that all ST398 MRSA isolates were detected in the period 2010–2013. Other molecular typing methods were also used, such SCC_*mec*_ analysis, *spa* typing and rep-PCR (Diversilab, bioMérieux). All these four techniques were in good agreement, with *spa* typing and rep-PCR being more discriminative than MLST and SCC_*mec*_ typing.

**Conclusions:**

This study is the first epidemiological study in France with extensive characterization of MRSA isolates associated with horse infections in stud farms. It shows that there is a significant increase of MRSA prevalence between 2007 and 2013, which mainly results from the spread of ST398 clones. It also highlights the importance of horses as a potential reservoir of important antimicrobial resistance genes.

## Background

Initially reported as a major cause of hospital-acquired infections in humans, methicillin-resistant *Staphylococcus aureus* (MRSA) has increasingly been reported as responsible for community-acquired infections as well as for infections in animals. Possible transmission of MRSA between humans and animals has raised concern about the role of animals as major reservoirs of MRSA clones involved in human infections [1–4]. Although MRSA strains are usually resistant to β-lactams through the acquisition of the *mec*A gene, a homolog gene (called *mecC*) has been recently reported both in animal and human populations [5–7]. It is well known that MRSA is responsible for a large variety of infections in numerous animals; however, specific studies in horses are scarce [6–9]. Indeed, some studies have demonstrated that horses are colonized and infected by MRSA clones that commonly belong to the sequence type (ST)8 and related STs within the clonal complex (CC)8 [6, 9]. More recently, studies from Europe and Canada reported horses to be colonized by MRSA clones belonging to ST398, designated livestock-associated (LA)-MRSA, which is primarily recognized as a colonizer of pigs and pig farmers [6]. LA-MRSA ST398 can be responsible for infections in humans in close contact with animals. Phenotypically, LA-MRSA ST398 is generally susceptible to antibiotics other than β-lactams even if it is characteristically resistant to tetracyclines [6]. Note that almost all equine MRSA isolates carry *mecA* while *mecC* has been rarely detected so far [6, 7]. From an epidemiological point of view, the prevalence of MRSA in horse infections has been poorly investigated in France [7, 10] and most importantly there is no data on nationwide molecular epidemiology.

The aim of the study was then 1) to evaluate the prevalence of MRSA isolated from horse clinical samples recovered between 2007 and 2013 in France, and 2) to extensively characterize phenotypically and genotypically this large collection of equine MRSA strains.

## Results

### Prevalence of MRSA isolates

**Fig. 1:**
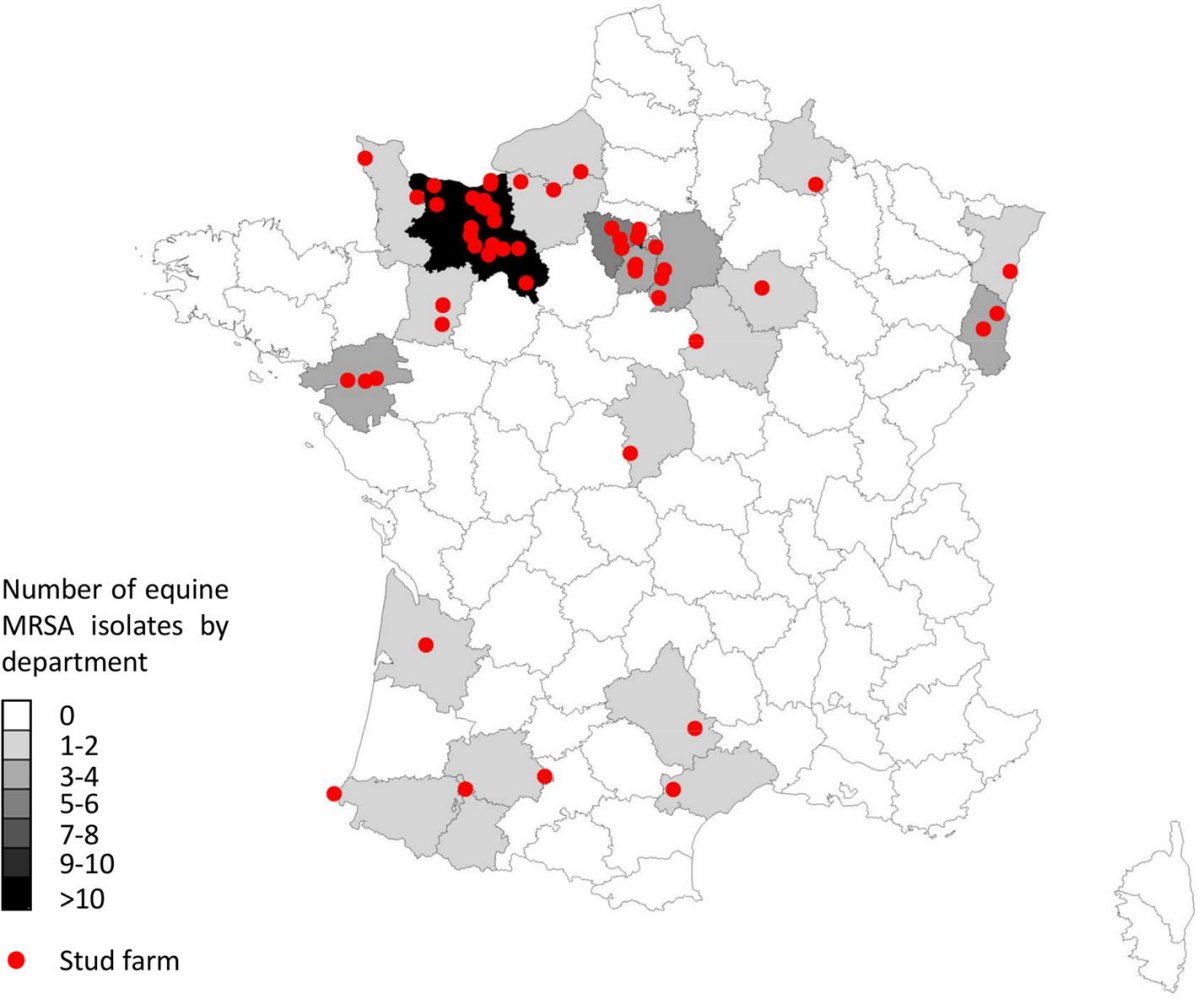
Map of France representing the distribution of stud farms and the number of MRSA isolates recovered from horses by department. The figure was obtained using the online map tool available at www.lion1906.com/Pages/francais/utile/outils.html#

From 2007 to 2013, the laboratory received 226,878 horse clinical samples with the recovery of 17,651 different bacterial isolates. *S. aureus* was the third most frequent bacterial species isolated (*n* = 1393; 7.9%) after group C streptococcus (*n* = 4510; 25.6%) and *Escherichia coli* (*n* = 3481; 19.7%). Out of the 1393 *S. aureus* horse isolates, 85 (6.1%) were categorized as MRSA (Table 1). Interestingly, the prevalence of MRSA significantly increased from 2007–2009 to 2010–2013 (0.7 vs. 9.5%, *P* <0.0001) (Table 1). They were recovered from different sources of infection: skin and soft-tissue (*n* = 39), genital tract (*n* = 20), respiratory tract (*n* = 8), bone and joint (*n* = 8) and others (*n* = 10). Note that MRSA isolates were collected from 56 different stud farms located in 24 different French departments (1 to 24 strains by department), mainly representing the Northwestern parts of France (Fig. 1). This roughly corresponds to the actual geographical distribution of stud farms with a high number of them in Normandy. No isolate harboured *pvl* and *tst* toxin genes (data not shown).

**Table 1:** Prevalence of MRSA from 2007 to 2013

Year	No. of *S. aureus*	No. of MRSA	% MRSA	Type of infection^a^					% ST8/ST398^b^
				SSTI	GTI	RTI	BJI	Others	
2007	163	2	1.2%	1	0	0	1	0	100/0
2008	198	2	1.0%	2	0	0	0	0	100/0
2009	190	0	0.0%	0	0	0	0	0	-
2010	226	15	6.6%	10	1	1	3	0	53/47
2011	253	22	8.7%	9	7	2	1	2	14/81
2012	185	24	12.9%	13	6	3	0	3	40/52
2013	178	20	11.2%	4	6	2	3	5	15/80
Total	1393	85	6.1%	39	20	8	8	10	

^a^ *SSTI* skin and soft-tissue infection, *GTI* genital-tract infection, *RTI* respiratory-tract infection, *BJI* bone and joint infection

^b^ *ST* sequence type

### Antimicrobial resistance profiles

Resistance to methicillin was associated with the presence of the *mecA* gene in 84 isolates (98.8%) while the *mecC* gene was detected in only one strain isolated in 2012 (1.2%) (Table 2). Note that this *mecC*-positive strain was susceptible to all other antibiotics (Table 2). A vast majority (83/85, 97.6%) were resistant to aminoglycosides with a KTG (kanamycin- tobramycin-gentamicin) phenotype, which was due to the presence of the *aac(6′)-aph(2″*) gene in all cases (Table 2). Concerning macrolide-lincosamide-streptogramin (MLS) antibiotics, 23 strains (27.1%) were resistant to erythromycin, most of them (20/23) being *erm*(C) positive with an inducible MLS_B_ resistance (Table 2). Almost all strains were resistant to both tested tetracyclines due to the presence of *tet*(M) only (81/85, 95.3%) or both *tet*(M) and *tet*(K) genes (2/85, 2.3%) (Table 2). Some strains (24/85, 28.2%) were resistant to fluoroquinolones, including 16 (66.7%) harbouring mutations in *gyrA* and/or *parC* quinolone resistance-determining regions (QRDRs) and 8 (33.3%) with putative active efflux (Table 2). Some isolates were categorized as intermediate/resistant to rifampin (24/85, 28.2%), cotrimoxazole (30/85, 35.3%) and chloramphenicol (23/85, 27.1%) (Table 2). Finally, all strains remained susceptible to glycopeptides, linezolid and fusidic acid. Note that the vast majority of the strains (83/85, 97.6%) was multiply resistant to at least three different classes of antibiotics.

**Table 2:** Antimicrobial resistance profiles and acquired mechanisms of resistance of the 85 MRSA isolates

Antibiotype^a^	Year of isolation (number of strains)	Antimicrobial resistance phenotype^b,c,d^	Acquired resistance gene(s)	Mutations in QRDR	
				*gyrA*	*parC*
**1**	2012 (1)	OXA	*mecC*		
**2**	2013 (1)	OXA, TE, MI	*mecA*, *tet*(M)		
**3**	2011 (1)	OXA, K, T, G, CIP	*mecA*, *aac(6′)-aph(2″)*	S84L	S80F, E84K
**4a**	2010 (1), 2011 (2)	OXA, K, T, G, **CIP**, TE, MI	*mecA*, *aac(6′)-aph(2″)*, *tet*(M)	-	-
**4b**	2010 (1)	OXA, K, T, G, **CIP**, TE, MI	*mecA*, *aac(6′)-aph(2″)*, *tet*(M), *tet*(K)	-	-
**5**	2011 (2), 2012 (1)	OXA, K, T, G, CIP, TE, MI	*mecA*, *aac(6′)-aph(2″)*, *tet*(M)	S84L	S80F
**6**	2010 (1)	OXA, K, T, G, **CIP**, TE, MI, RA, SXT	*mecA*, *aac(6′)-aph(2″)*, *tet*(M)	-	-
**7**	2012 (1)	OXA, K, T, G, CIP, TE, MI, RA, SXT, C	*mecA*, *aac(6′)-aph(2″)*, *tet*(M)	-	S80F
**8**	2012 (1)	OXA, K, T, G, CIP, TE, MI, SXT	*mecA*, *aac(6′)-aph(2″)*, *tet*(M)	S84L	S80F, E84D
**9**	2010 (1)	OXA, K, T, G, **CIP**, TE, MI, RA, SXT	*mecA*, *aac(6′)-aph(2″)*, *aph(3′)-IIIa*, *ant(4′)-Ia*, *tet*(M)	-	-
**10**	2013 (1)	OXA, K, T, G, E(c), **CIP**, TE, MI, SXT	*mecA*, *aac(6′)-aph(2″)*, *tet*(M)*, erm*(B)	-	-
**11**	2013 (1)	OXA, K, T, G, E(c), TE, MI, RA, SXT	*mecA*, *aac(6′)-aph(2″)*, *tet*(M)*, erm*(C), *msr*(A)		
**12a**	2012 (7), 2013 (2)	OXA, K, T, G, E(i), CIP, TE, MI, RA, SXT, C	*mecA*, *aac(6′)-aph(2″)*, *tet*(M)*, erm*(C)	-	S80F
**12b**	2013 (1)	OXA, K, T, G, E(i), CIP, TE, MI, RA, SXT, C	*mecA*, *aac(6′)-aph(2″)*, *tet*(M)*, erm*(C), *msr*(A)	-	S80F
**13**	2013 (1)	OXA, K, T, G, E(i), TE, MI	*mecA*, *aac(6′)-aph(2″)*, *tet*(M)*, erm*(C)		
**14**	2007 (2), 2010 (1), 2011 (1)	OXA, K, T, G, E(i), TE, MI, RA, SXT	*mecA*, *aac(6′)-aph(2″)*, *tet*(M)*, erm*(C)		
**15**	2008 (1)	OXA, K, T, G, E(i), TE, MI, RA, SXT, C	*mecA*, *aac(6′)-aph(2″)*, *tet*(M)*, erm*(C)		
**16**	2010 (3), 2011 (1), 2012 (1)	OXA, K, T, G, E(i), TE, MI, SXT	*mecA*, *aac(6′)-aph(2″)*, *tet*(M)*, erm*(C)		
**17**	2008 (1), 2011 (1)	OXA, K, T, G, E(i), TE, MI, SXT, C	*mecA*, *aac(6′)-aph(2″)*, *tet*(M)*, erm*(C)		
**18**	2010 (1)	OXA, K, T, G, E, **CIP**, TE, MI, RA	*mecA*, *aac(6′)-aph(2″)*, *aph(3′)-IIIa*, *tet*(M)*, msr*(A)	-	-
**19a**	2010 (2), 2011 (10), 2012 (11), 2013 (10)	OXA, K, T, G, TE, MI	*mecA*, *aac(6′)-aph(2″)*, *tet*(M)		
**19b**	2010 (1)	OXA, K, T, G, TE, MI	*mecA*, *aac(6′)-aph(2″)*, *ant(4′)-Ia*, *tet*(M)		
**20a**	2011 (1), 2012 (2), 2013 (2)	OXA, K, T, G, TE, MI, C	*mecA*, *aac(6′)-aph(2″)*, *tet*(M)		
**20b**	2013 (1)	OXA, K, T, G, TE, MI, C	*mecA*, *aac(6′)-aph(2″)*, *tet*(M), tet(K)		
**21**	2010 (1)	OXA, K, T, G, TE, MI, RA	*mecA*, *aac(6′)-aph(2″)*, *tet*(M)		
**22**	2010 (2)	OXA, K, T, G, TE, MI, RA, C	*mecA*, *aac(6′)-aph(2″)*, *tet*(M)		
**23**	2011 (1)	OXA, K, T, G, TE, MI, RA, SXT	*mecA*, *aac(6′)-aph(2″)*, *tet*(M)		
**24**	2012 (1)	OXA, K, T, G, TE, MI, SXT	*mecA*, *aac(6′)-aph(2″)*, *tet*(M)		

^a^Strains were classified according to their antimicrobial resistance phenotypes (1 to 24). Strains exhibiting identical antimicrobial resistance phenotypes but different genotypes were differentiated as 4a/4b, 12a/12b, 19a/19b, and 20a/20b

^b^Resistance to: *C* chloramphenicol, *E* erythromycin, *G* gentamicin, *K* kanamycin, *MI* minocycline, *OXA* oxacillin, *CIP* ciprofloxacin, *RA* rifampin, *SXT* cotrimoxazole, *TE* tetracycline, *T* tobramycin

^c^E(i), inducible MLS_B_ resistance phenotype; E(c), constitutive MLS_B_ resistance phenotype

^d^ **CIP**, fluoroquinolone resistance putatively due to an active efflux (≥2-fold decrease in MIC of ciprofloxacin in the presence of 10 μg/ml of reserpine)

### Analysis of clonal populations

**Fig. 2:**
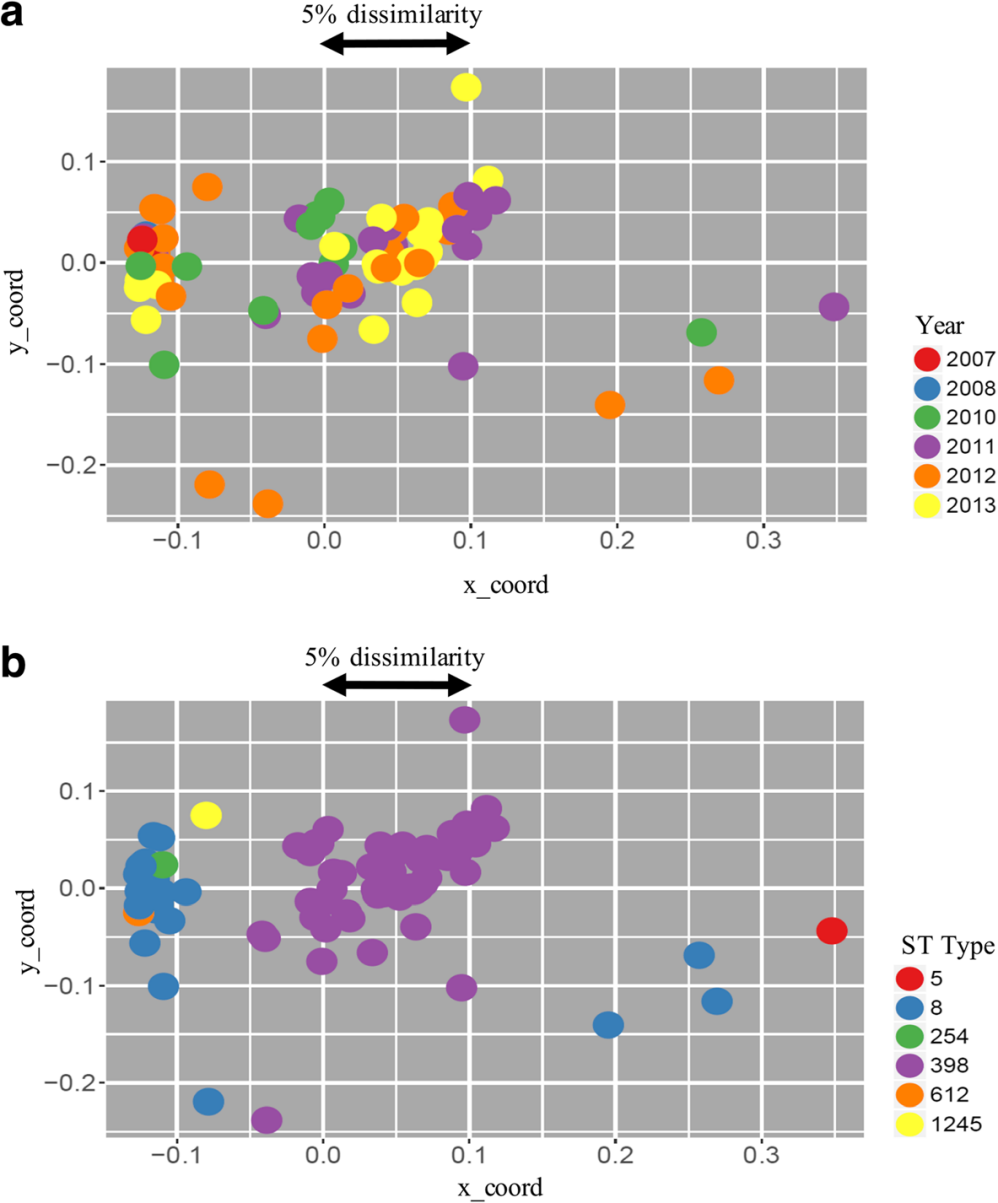
Scatterplots derived from Diversilab data of the MRSA showing year of isolation (**a**) and ST type (**b**). The scale indicates the dissimilarity between strains for the X and Y axes. The graphs were obtained using the using R software and ggplot2 package

According to phenotypic and genotypic antimicrobial resistance profiles, 24 different profiles were distinguished (Table 2). MLST revealed that the majority of MRSA isolates belonged to two main STs: ST8 (28/85, 32.9%) and ST398 (53/85, 62.4%) (Table 3). Interestingly, all ST398 MRSA isolates were detected in 2010–13 (Tables 1 and 3). By SCC_*mec*_ analysis, the SCC_*mec*_ type IVd was identified in 27/28 (96.4%) ST8 isolates while the SCC_*mec*_ type IVa found in 52/53 (98.1%) ST398 isolates (Table 3). The *spa* typing differentiated the MRSA collection into 15 distinct *spa* types. The *spa* types found among the ST8 strains were as follows: t064 (2/28, 7.1%), t394 (16/28, 57.1%), t451 (3/28, 10.7%), t13440 (6/28, 21.4%) and t5488 (1/28, 3.6%) (Table 3). Among ST398 strains, the *spa* type t011 was largely predominant (44/53, 83.0%) followed by t108 (1/53, 1.9%), t1255 (3/53, 5.7%), t899 (2/53, 3.8%), t1451 (1/53, 1.9%) and t2346 (2/53, 3.8%) (Table 3). The rep-PCR technique delineated 38 different clusters (using a similarity index of 98.38% as determined by the Diversilab software) with 66 isolates being grouped in ≥2-isolate clusters and 19 isolates corresponding to singletons (Fig. 1a and Fig. 2a). There was a good agreement between MLST and rep-PCR with the latter method being much more discriminative, especially within the ST398 cluster (Fig. 1b and Fig. 2b). Then, it was possible to distinguish some specific lineages related to certain geographical regions. In addition, rep-PCR had the advantage to dissect the genetic relatedness of ST398 clones since these isolates are not typeable by pulsed-filed gel electrophoresis (PFGE) using *Sma*I [11].

**Table 3:** Comparison of the results obtained by MLST, SCC_*mec*_ analysis and *spa* typing for the 85 MRSA horse isolates

Year of isolation	Sequence type [ST] (no.)	*spa* type (no.)	SCC_*mec*_ type (no.)
2007	8 (2)	t394 (2)	IVd (2)
2008	8 (2)	t064 (1), t394 (1)	IVd (2)
2010	8 (8) 398 (7)	t394 (1), t451 (3), t5488 (1), t13440 (3) t011 (7)	II (1), IVd (7) IVa (6), V (1)
2011	5 (1) 8 (3) 398 (17)	t777 (1) t394 (1), t13440 (2) t011 (14), t1255 (2), t2346 (1)	VI (1) IVd (3) IVa (17)
2012	8 (10) 254 (1) 398 (13) 1245^a^ (1)	t064 (1), t394 (8), t13440 (1) t009 (1) t011 (10), t899 (1), t1255 (1), t2346 (1) t6220 (1)	IVd (10) nt^b^ IVa (13) nt^b^
2013	8 (3) 398 (16) 612 (1)	t394 (3) t011 (13), t108 (1), t899 (1), t1451 (1) t064 (1)	IVd (3) IVa (16) IVd (1)

^a^ *mecC*-positive strain

^b^ *nt* not typeable

## Discussion

In this study, we have showed that the prevalence of MRSA strains isolated from horse infections before 2011 was low (~2%) in France, with ST8 being predominant during this period. The prevalence has since increased from 2011 to reach about 10%, and ST398 has become the predominant MRSA lineage. These data on MRSA prevalence in equine infections are in agreement with those observed in other European veterinary hospitals: 22% in Switzerland [12] and 19% in Germany [13]. However, it is important to note that MRSA isolates in our study were recovered in stud farms and not in veterinary hospitals. Indeed, the lower prevalence in France observed in our study may be explained by the fact that these samples are from stud farms that are exposed to different environmental conditions and risk factors as compared to veterinary hospitals. Although horses were generally hosted in French stud farms geographically distant, these results suggest a national diffusion of clonally related MRSA ST8 and ST398 isolates.

The *mec*A-carrying ST8 and ST398 have been reported as the major strains that infected or colonized horses [13, 14]. Colonization and infection of horses in the USA and Canada generally involve ST8-type MRSA isolates, classified by PFGE as Canadian MRSA-5 or USA500 [6]. ST8 is infrequently found in humans and is among STs isolated from horses of the clonal complex (CC) 8. ST8-type MRSA has also been recently isolated in Australian horses [15]. In France, only one molecular study has been performed on MRSA isolated in horses [10]. In this study, only three MRSA were identified among 59 *S. aureus* equine isolates and all belonged to the ST8 [10]. ST398-type MRSA is a non-CC8 clone initially report in France in pigs [6], which is currently spreading in horse clinics in Europe and North America [10, 12, 16]. Even if it is characteristically resistant to tetracyclines, this emerging ST is generally susceptible to other antibiotics, which was quite different from what we observed in our study. Indeed, we found that 52 out of 53 (98.1%) ST398 strains were multiply resistant to at least three classes of antibiotics. Even though multidrug resistance in MRSA is de facto defined as MDR [17], it is important to highlight the very high proportion (97.6%) of strains exhibiting resistant to three-to-eight antimicrobial categories. Although ST398 is associated with livestock, sporadic cases, and outbreaks in equine hospitals, colonization of horses and associated personnel have been reported in Europe [6, 11, 18, 19]. Recently, a suspected transmission of MRSA ST398 from a horse to a Dutch girl, which resulted in a foot infection, has been reported [20].

Among other STs, ST612, which belong to the CC8, was recently described in horses and seems to be strongly associated with equine practice veterinarians [21] while ST5, described in pets, swine and poultry [6], was recently reported in horses in Japan [22]. Finally, some authors have recently characterized in animal MRSA isolates belonging to CC130 (ST1245), the most prevalent CC among *mecC*-positive strains [7, 23, 24].

## Conclusions

In conclusion, this first epidemiological survey conducted in France has shown an increase in the prevalence of MRSA isolates associated with horse infections since 2010 in stud farms, which is in part related to the emergence of clonally-related ST398 MRSA isolates. Since this new ST398 type is known to cause outbreaks in horses and to colonize/infect humans, hygiene measures and appropriate antimicrobial use should be maintained and reinforced in order to limit the transmission of *S. aureus* between horses as well as between horses and humans.

## Methods

### Bacterial isolates and antimicrobial susceptibility testing

The regional veterinary laboratory of Normandy (LABÉO) is specialized in the analysis of specimens from infected horses and receives clinical samples from numerous stud farms located in various regions of France. From January 2007 to December 2013, all non-duplicate clinical isolates of *S. aureus* were prospectively studied.

Over this period, all MRSA horse isolates were further characterized. Species-level identification was performed using the MALDI-TOF mass spectrometry technology (Microflex; Bruker Daltonics, Bremen, France) and, if necessary, by amplification of the *S. aureus*-specific *nuc* gene, as previously described [24]. Methicillin resistance was confirmed by the detection of both *mecA* and *mecC* genes, as previously described [5, 25, 26].

Antimicrobial susceptibility testing was performed using the agar diffusion method, as recommended by the Antibiogram Committee of the French Society for Microbiology (www.sfm-microbiologie.org/). The following antibiotics were tested: oxacillin, kanamycin, tobramycin, gentamicin, erythromycin, clindamycin, pristinamycin, ciprofloxacin, vancomycin, teicoplanin, linezolid, tetracycline, minocycline, rifampin, cotrimoxazole, chloramphenicol and fusidic acid. A double-disc diffusion test (D-test) was used to detect the inducible MLS_B_ resistance phenotype.

### PCR and molecular typing

Genes conferring resistance to MLS [*erm*(A), *erm*(B), *erm*(C), and *msr*(A)], aminoglycosides [*aph(3′)-IIIa*, *ant(4′)-Ia*, and *aac(6′)-aph(2″*)] and tetracyclines [*tet*(M) and *tet*(K)] were screened by PCR, as previously described [27–29]. Mechanisms of fluoroquinolone resistance were studied by sequencing QRDRs of *gyrA* and *parC* genes [30] and by determining MICs of ciprofloxacin with or without reserpin (10 μg/ml). Both *pvl* and *tst* genes coding for Panton-Valentine leukocidin (PVL) and toxic shock staphylococcal toxin (TSST-1), respectively, were screened by PCR as previously described [31–33].

For molecular typing, four different techniques were used for all the strains. The MLST was performed as previously described [34] using the MLST database (http://saureus.mlst.net/). The *spa* typing was carried out as previously described [35] using the Ridom StaphType software (Ridom GmbH, Würzburg, Germany). The typing of staphylococcal cassette chromosome *mec* element (SCC*mec*) was performed according to *mec* and *ccr* complexes previously defined [36]. Genetic relatedness was determined by rep-PCR using the semi-automated Diversilab system (bioMérieux, Marcy l’étoile, France) [14, 37].

### Statistical analysis

The Fisher’s exact test was used to compare categorical variables. *P* values <0.05 were considered to be statistically significant. All tests were 2 tailed. Statistical tests were performed using GraphPad Prism, version 6.

## Abbreviations

CC: Clonal complex
MALDI-TOF: Matrix-assisted laser desorption/ionization-time-of-flight
MIC: Minimal inhibitory concentration
MLS: Macrolides-lincosamides-streptogramins
MLST: Multi-locus sequence typing
MRSA: Methicillin-resistant *Staphylococcus aureus*
PFGE: Pulsed-field gel electrophoresis
PVL: Panton-Valentine leucocidin
QRDR: Quinolone-resistance determining region
SCC*mec*: Staphylococcal cassette chromosome *mec* element
ST: Sequence type
TSST-1: Toxic shock staphylococcal toxin
